# Research on autonomous route generation method based on AIS ship trajectory big data and improved LSTM algorithm

**DOI:** 10.3389/fnbot.2022.1049343

**Published:** 2022-11-24

**Authors:** ChangXi Zhuang, Chao Chen

**Affiliations:** Maritime School, Zhejiang Ocean University, Zhoushan, China

**Keywords:** AIS ship trajectory big data, ship intelligence, route autonomous generation, clustering algorithm, LSTM

## Abstract

The autonomous generation of routes is an important part of ship intelligence and it can be realized by deep learning of the big data of automatic identification system (AIS) ship trajectories. In this study, to make the routes generated by long short-term memory (LSTM) artificial neural network more accurate and efficient, a ship route autonomous generation scheme is proposed based on AIS ship trajectory big data and improved multi-task LSTM artificial neural network. By introducing an unsupervised trajectory separation mechanism into LSTM, a fast and accurate separation of trajectories with similar paths is realized. In the process of route generation, first of all, a clustering algorithm is used to cluster the trajectories in massive AIS data according to the density of trajectory points, so as to eliminate the trajectories in the routes that do not belong to the target area. Furthermore, the routes are classified according to the type of ships, and then the classified trajectories are processed and used as datasets. Based on these datasets, an improved LSTM algorithm is used to generate ship routes autonomously. The results show the improved LSTM works better than LSTM when the generated route trajectories are short.

## Introduction

With the development of the global shipping economy, ships are gradually developing in the direction of large scale, high speed, and intelligence, and more attention has been paid to the safety and economy of ship navigation which depends to a large extent on the adoption of a correct and reasonable route. As the first priority of ship navigation planning, route planning design is an important and tedious task (Yao, 2019). The automatic identification system (AIS) of ship is a kind of navigation aid equipment and is used for maritime safety and communication between ships as well as between ships and shore. AIS can broadcast the dynamic information of ships such as ship position, ship speed, and heading to other ships and shore stations in the nearby waters in combination with the static information of ships such as ship name, call sign, and draft (Feng et al., 2021). The historical voyage big data of navigation ships can be obtained by collecting AIS data. Through the data mining of the historical trajectory of ships' navigation in a certain area, the navigation patterns of ships can be analyzed. Furthermore, based on this, reasonable routes can be recommended for ships sailing in this area (Zhang et al., 2015). How to use the AIS big data of ships to improve the intelligence of route planning design and finally realize the autonomous generation of the route is a challenging topic.

At present, the route generation technology can be mainly divided into two classes. One is that the relevant experts and technicians draw the route on the paper chart manually, and the other is to use the evolutionary algorithms to automatically generate the navigation route of the ship (Zeng and Ito, 2001; Shen et al., 2019). The route designed by evolutionary algorithms can greatly reduce the workload of the crew and enhance the intellectualization of ship operation. Moreover, evolutionary algorithms can be divided into two types, one is according to the sea depth, weather, wind direction, wind speed, and other factors, through computer simulation to find the optimal path algorithm (Gasparetto et al., 2015; Dai et al., 2019). The other is based on AIS big data combined with a deep learning algorithm to achieve autonomous route generation (Lv et al., 2018; Lazarowska, 2020). Compared with manual route drawing, the former reduces the complexity of route design but often falls into a local optimal solution. In contrast, deep learning algorithms have been widely used in image recognition, language processing, traffic flow prediction, and other fields, and the generation technology of vehicle driving recommended routes based on big data is also becoming mature (Arel et al., 2010; Islam et al., 2016; Zhou et al., 2017). Against this background, this study proposes a method to classify ship trajectory according to the navigation routes by adding an unsupervised clustering layer based on long short-term memory (LSTM) deep learning algorithm with the historical trajectory of ships as the dataset and finally realizing the autonomous generation of ship routes. The algorithm can not only make the route design more intelligent and convenient but can also improve the navigation safety to a certain extent.

The trajectory clustering algorithm is an unsupervised learning algorithm that can classify trajectories according to the similarity of the trajectories (Pauletic et al., 2019). According to the different measurement methods, the related studies are mainly divided into two categories, one is based on trajectory points, and the other is based on trajectory segments. In the research on clustering algorithm based on trajectory points, Morris and Trivedi (2009) compared various trajectory clustering algorithms and their characteristics on different datasets, but it has not been verified in practical applications. Piciarelli et al. (2005) proposed a real-time trajectory clustering algorithm based on video datasets, which can obtain valuable data from higher-level anomaly detection modules. Zhao et al. (2017) proposed a hierarchical clustering method and an adaptive statistical method to solve the problem of uneven distribution of ship trajectories, but the method in a more complex environment was not considered. The clustering method based on trajectory points can deal with large trajectory data, but it ignores the space-time correlation between points and is not sensitive to abnormal trajectory points. In contrast, in the research on clustering algorithm based on trajectory segment, Lee et al. (2007) proposed the trajectory-based Hausdorff distance method that calculates the distance between trajectory segments in terms of parallel distance, vertical distance, and angular distance, and its results are more accurate.

Lin and Su (2008) proposed a one-way distance method based on the spatial shape of a moving object trajectory that only pays attention to the similarity of trajectory space shape and ignores time, speed, direction, and other attributes. The clustering algorithm based on trajectory segment has a good consideration for the accuracy of the trajectory, but the time complexity is relatively high, which is not conducive to use when the data volume is large. From the two kinds of clustering algorithm, the current clustering algorithms cannot take into account both trajectory integrity and efficiency, which is very difficult to handle the huge AIS big data.

Long short-term memory artificial neural network is a kind of recurrent neural network (RNN), which is used to solve the gradient vanishing problem of RNN. Many scholars use LSTM to generate trajectories. Xing et al. (2019) divided the vehicles into different types according to their driving styles and predicted the trajectory errors at different driving times. However, it did not carry out the comparison of the accuracy of the generated trajectories with different lengths. Xue et al. (2020) used LSTM to predict pedestrian trajectory, but it has not been confirmed whether it was effective in complex scenarios. Kong et al. (2019) proposed a new RNN-based default logic for path planning with graph-based search algorithms and optimization methods among existing urban road planning methods. Lin et al. (2021) proposed STA-LSTM to improve the interpretability of vehicle trajectory prediction. The current research on LSTM trajectory generation mainly focuses on the improvement of the model, ignoring the influence of the accuracy of the trajectory dataset on the final prediction results. The trajectories of vehicles on roads are relatively neat, while the trajectories of ships at sea are sparse, and the trajectories of different ship types vary greatly. Therefore, how to reduce the trajectory generation error on the sea is an urgent problem to be solved.

According to the problems in the above research, this work makes the contributions as follows:

(1) Using the actual latitude and longitude to calculate the distance instead of the Euclidean distance in the clustering algorithm makes the clustering algorithm more accurate in dealing with ship trajectories to produce more accurate results.
(2) In the actual study, it is found that the sea surface paths are sparse and the complete route trajectories are more difficult to obtain. To address the problem of small trajectory samples, this study proposes a random dilution of high-density routes to obtain more samples.
(3) According to the AIS trajectory clustering problem, this work uses the k-means algorithm to cluster the target area. Then, a layer of unsupervised trajectory clustering layer is customized on top of the LSTM algorithm, and the adaptive DBSCAN clustering algorithm mentioned by Zhao et al. (2017) is fused into the unsupervised trajectory clustering layer. This study also widens the network width of the LSTM so that the LSTM can generate trajectories for multiple types of ships simultaneously. Moreover, this study compares the errors when generating trajectories of different lengths to prove the effectiveness of this study.

In this study, the western route with large navigable volume and complicated sea conditions in the Zhoushan sea area is selected as the research target area, and the specific route between Cezi Island and Liuheng Island in the western route is selected as the specific route planning target route, which has certain practical significance. The general arrangement of this study is as follows. The “Related principles” section introduces the relevant principles involved in study work and the improvement of the relevant principles. The “Production of datasets” section introduces the production process of the dataset. The “Route generation” section describes the experimental process and the comparison of the results of different models. The “Conclusion” section summarizes this experiment.

## Related principles

### Clustering algorithm

(1) K-means algorithm

The k-means algorithm is a division-based clustering method that needs to specify the clusters (K-values) during clustering, the number of clusters will affect the final clustering effect, and cross-validation can be used to select an appropriate K-value for clustering unknown data (Nie et al., 2022). The K-means algorithm has the following main steps:

**Step 1**. Let the trajectory data be ***D*** **=** **{** ***x***_***1***_**,*****x***_***2***_**,...,*****x***_***m***_**}**, the number of clusters is ***k***, and the maximum number of iterations is ***N***.

**Step 2**. The output cluster is ***C*** **=** **{*****C***_***1***_**,*****C***_***2***_**,...,*****C***_***k***_ **}**.

**Step 3**. Initialize ***C***_***i***_ **=** ∅, and choose suitable ***k*** points in D at random as the initial center of mass *μ* = (*μ*_***1***_,*μ**_2_*****,...**,*μ*_***k***_).

**Step 4**. Calculate the distance between each sample of D in the trajectory data and each centroid, and *y*_*j*_ denotes any trajectory point within the cluster:


(1) $$\begin{array}{l} dist\;(x_{i},y_{j})\;=\;||x_{i}-\mu_{j}|{|}^{2},\;i\;=\;1,2,...,n;j\;=\;1,2,...,k. \end{array}$$

**Step 5**. Assign the trajectory point *x*_*i*_ into the nearest clusters according to the distance calculated in the previous step, and **C**_**λ**_**i**__**=** **C**_**λ**_**i**__⋃ {**x**_**i**_} is updated.

**Step 6**. Update the initial center of mass of each cluster:


(2) $$\begin{array}{l} \mu_{j}^{{}^{\prime }}=\frac{1}{\left|C_{j}\right|}\underset{x\in |C_{j}|}{\sum }x \end{array}$$

**Step 7**. Repeat steps 4, 5, and 6 until the initial center of mass no longer changes.

(2)Adaptive DBSCAN

The Adaptive DBSCAN algorithm is a clustering algorithm that improves the DBSCAN algorithm. It improves the characteristics of the DBSCAN algorithm that requires manual input of parameters, introduces the concept of density threshold, and uses the KNN algorithm to determine the optimal parameters automatically. The specific steps are as follows:

**Step 1**. The density threshold is defined first, i.e.,


(3) $$\begin{array}{l} Density=\frac{MinPts}{\pi •Eps^{2}} \end{array}$$

**Step 2**. *MinPts* is the specified minimum number of trajectory points and *Eps* is the specified radius. On the premise that the number of clusters in the clustering result is correct, the smaller the density threshold, the better the clustering effect. The following equation is used to calculate the distance between the points:


(4) $$D_{n\times n}\;=\;\{Dist(i,j)|1\leq i\leq n,1\leq j\leq n\}$$

where ***n*** is the number of trajectory points, and *Dist*(*i, j*) is the Euclidean distance between the two trajectory points. The elements in ***D*** are sorted in ascending order for each row. All data points in the kth column are the K-nearest neighbor distance vector *D*_*k*_. After averaging, we can get $D_{k}$, which is calculated for all columns to obtain the list of *Eps* parameters:


(5) $$\begin{array}{l} D_{Eps}\;=\;\{D_{k}|1\leq K\leq n\} \end{array}$$

**Step 3**. The definition of *MinPts* is as follows:


(6) $$\begin{array}{l} MinPts\;=\;\frac{1}{n}\overset{n}{\underset{i=1}{\sum }}P_{i} \end{array}$$

where *P*_*i*_ is the number of *Eps* domain objects of the ith object, and n is the number of trajectory points.

**Step 4**. Input the parameters in *D*_*Eps*_ and the corresponding *MinPts* into the DBSCAN algorithm. The number of clusters ***N*** under different ***K*** values can be obtained. In addition, if the number of clusters ***N*** is unchanged for more than three consecutive times, the maximum ***K*** value corresponding to the number of clusters ***N*** is determined as the optimal ***K*** value, and then the *Eps* and *MinPts* values corresponding to the optimal ***K*** value are determined as the optimal parameters.

The distance calculated in formula (4) is Euclidean distance, which is not applicable to the distance of the trajectory points of ships but it uses the actual latitude and longitude instead. Let the latitude and longitude of point A and point B be (*x*_*i*_,*y*_*i*_) and (*x*_*j*_,*y*_*j*_), respectively, and the formula is as follows:


(7) $$Dist(i,j)\;=R[\cos(x_{i}-x_{j})\cos y_{i}\cos y_{j}+\sin y_{i}\sin y_{j}]$$

where R is the radius of the Earth.

### Improved LSTM algorithm

**Figure 1:**
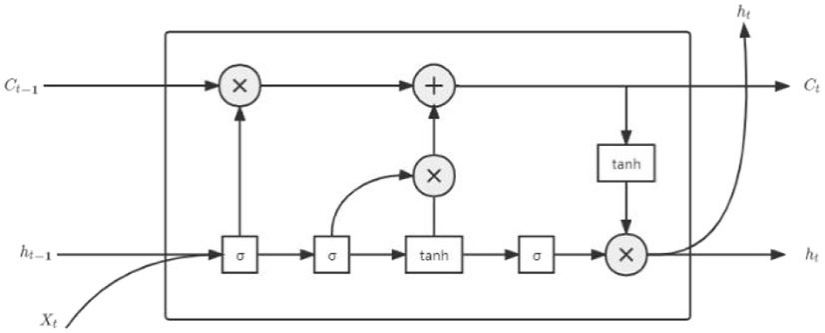
Structure diagram of LSTM neuron.

Long short-term memory is a special RNN, which is used to solve the problem of gradient vanishing and gradient exploding in traditional RNN. Compared with the ordinary RNN, LSTM solves the long-term dependence on historical data, which greatly improves the effect of neural network in solving regression problems (Zaremba et al., 2014). LSTM is also widely used in language recognition, text classification, stock forecasting, and other fields (Chakraborty et al., 2020). The structure of LSTM is shown in Figure 1.

In the figure, σ represents the sigmoid layer, whose values range is [0,1], where 0 means all forgotten and 1 means all remembered; tanh is the activation function; *h*_*t*_ represents the hidden layer at moment t; *X*_*t*_ represents the input route trajectory at moment t; *C*_*t*_ represents the route trajectory information at moment t. The LSTM is mainly controlled by input gate, output gate, and forget gate for the cell, and the principle is as follows.

Forget gate:


(8) $$\begin{array}{l} f_{t}\;=\;\sigma \left(W_{f}•\left[h_{t-1,}x_{t}\right]+b_{f}\right) \end{array}$$

Input gate:z


(9) $$\begin{array}{l} i_{t}\;=\;\sigma \left(W_{i}•\left[h_{t-1},x_{t}\right]+b_{i}\right) \end{array}$$

(10) $$\begin{array}{l} {\tilde{C}}_{t}\;=\;tanh\;(W_{c}•[h_{t-1},x_{t}]+b_{c}) \end{array}$$

(11) $$\begin{array}{l} C_{t}\;=\;f_{t}•C_{t-1}+i_{t}•\tilde{C_{t}} \end{array}$$

Output gate:


(12) $$\begin{array}{l} O_{t}\;=\;\sigma \;(W_{o}\left[h_{t-1},x_{t}\right]+b_{o}) \end{array}$$

(13) $$\begin{array}{l} h_{t}\;=\;O_{t}•tanh\left(C_{t}\right) \end{array}$$

where *W*_*f*_ denotes the weight matrix in the forget gate, *b*_*f*_ denotes the bias matrix in the forget gate, $\tilde{C_{t}}$ denotes the candidate vector at moment t, *i*_*t*_ denotes the input route trajectory at moment t, *O*_*t*_ denotes the output at moment t, and *f*_*t*_ denotes the forget information at moment t.

Long short-term memory is a chain structure; in the initial state, values of *h*_0_ and *C*_0_ are 0; and at this time, the history information is empty. In addition, when the input route trajectory information *X*_1_ passes through the first cell, *h*_*t*−1_ and *C*_*t*−1_ are generated. In the next cell, *h*_*t*−1_ and *x*_*t*_ are output through the *f*_*t*_ of the sigmoid layer to decide which route information to forget. Then, the input gate generates *i*_*t*_ and $\tilde{C_{t}}$ according to *h*_*t*−1_ and *x*_*t*_, and the current state *C*_*t*_ can be obtained by multiplying the two parts. The output gate outputs *O*_*t*_ through the sigmoid layer according to *h*_*t*−1_ and *x*_*t*_, and the final output *h*_*t*_ can be obtained by multiplying *O*_*t*_ and the activation tanh(*C*_*t*_). Then, output *h*_*t*_ can be used as the input to the next cell and so on (Gers et al., 2000). During the operation of the LSTM, *h*_*t*−1_ represents the short-term memory, which is updated at each moment, while *C*_*t*−1_ represents the long-term memory, which can save the route trajectory information for a certain time interval, but less than the long-term memory, so the LSTM is called the long- and short-term memory neural network (Huang et al., 2015). Equations (2-1) to (2-6) can be simplified as


(14) $$\begin{array}{l} \left[\begin{array}{c} \begin{array}{c} \tilde{C_{t}}\\ O_{t} \end{array}\\ i_{t}\\ f_{t} \end{array}\right]\;=\;\left[\begin{array}{c} \begin{array}{c} \tanh\\ \sigma \end{array}\\ \sigma \\ \sigma \end{array}\right]\;(W\left[\begin{array}{c} x_{t}\\ h_{t-1} \end{array}\right]+b) \end{array}$$

(15) $$\begin{array}{l} C_{t}\;=\;f_{t}•C_{t-1}+i_{t}•\tilde{C_{t}} \end{array}$$

(16) $$\begin{array}{l} h_{t}\;=\;O_{t}•tanh\;(C_{t}) \end{array}$$

**Figure 2:**
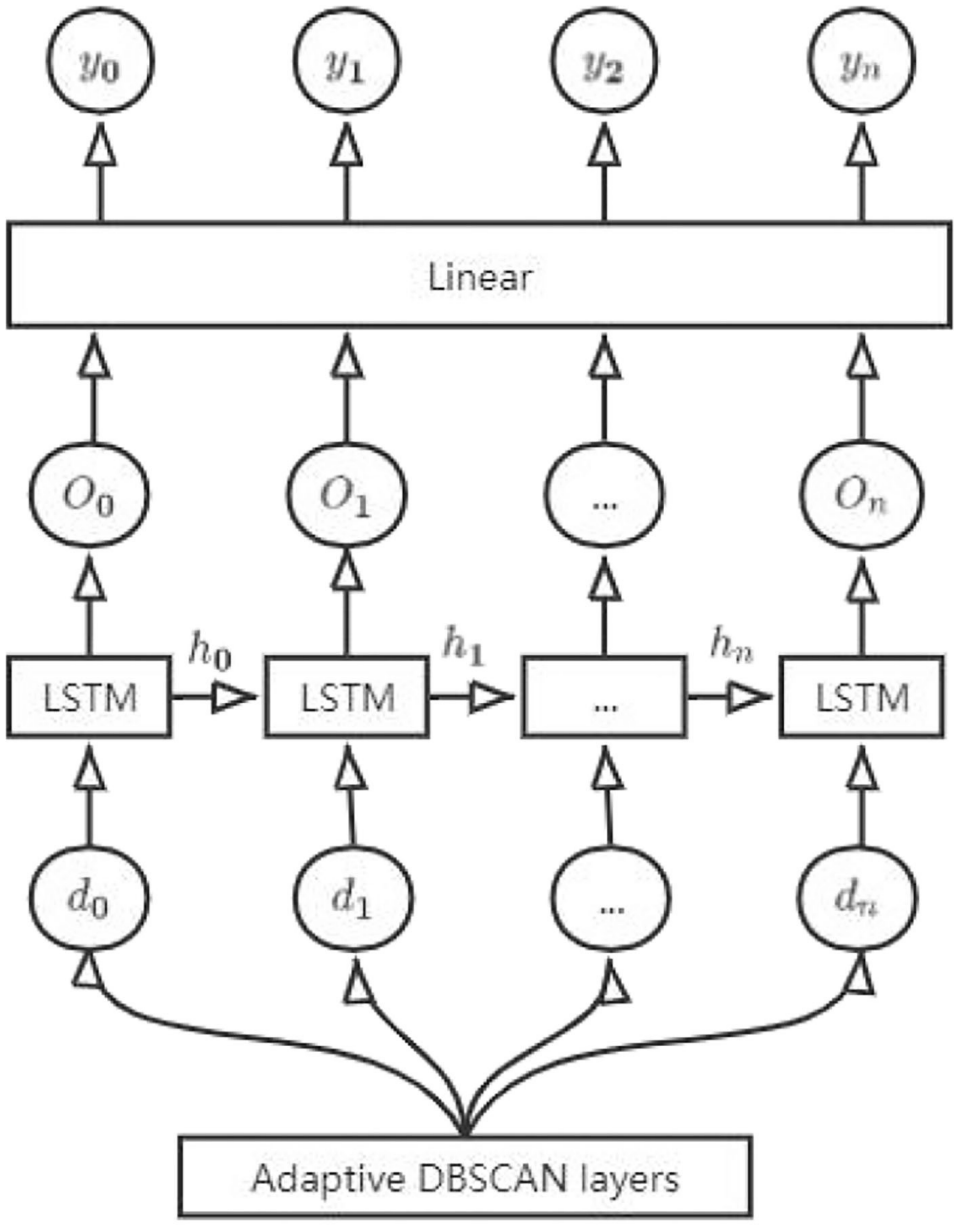
Structure diagram of the improved LSTM.

The improved LSTM adds a clustering layer on top of the LSTM. The input trajectories are not directly entered into the LSTM layer but are first classified through the clustering layer. The number of trajectories *P* for each class is counted according to the MMSI number, and the classes exceeding *P* are input into the LSTM layer. The improved LSTM is shown in Figure 2. In the figure, *y*_*t*_ denotes the output route trajectory at moment t, and n denotes the length of the sequence.

The set of classified data is denoted as ***M***** = {*****m***_**1**_**,*****m***_**2**_**,…,*****m***_***N***_**}**, and d is denoted as the class when *M*_*i*_ ≥ *P*. Then, Equation 9 should be


(17) $$\begin{array}{l} i_{t}\;=\;\sigma \left(W_{i}•\left[h_{t-1},d_{t}\right]+b_{i}\right) \end{array}$$

### Evaluation of LSTM algorithm

In this study, two evaluation methods are used to evaluate the performance of the model.

(1) Average absolute error


(18) $$MAE\;=\;\frac{1}{N}\sum_{t-1}^{N}|y(t)-\hat{y}\left(t)\right|$$

(2) Root mean square error (RMSE)


(19) $$RMSE\;=\;\sqrt{\frac{1}{N}\sum_{t-1}^{N}{(y(t)-\hat{y}\left(t)\right)}^{2}}$$

where N is the total number of routes, *y*(*t*) is the actual value of the route at time t, and ŷ(*t*) is the predicted value of the route at time t. RMSE has a faster convergence speed and more accurate results, while MAE has strong robustness and is insensitive to outliers. When the error value is smaller, the trajectory is more similar, and the prediction accuracy of the model is higher (Felix and Schmidhuber, 2000).

### Route generation process

The autonomous route generation technique designed in this work mainly includes the following steps.

(1) Determine the departure point and destination area of the route.
(2) Screen and clean the AIS data in the area.
(3) The AIS trajectories in the area are clustered by the k-means clustering algorithm, and the clusters that the routes pass through are reserved.
(4) Interpolate routes with missing trajectories and sparse dense routes into multiple trajectory samples.
(5) Compare the model before and after improvement and analyze the results.

**Figure 3:**
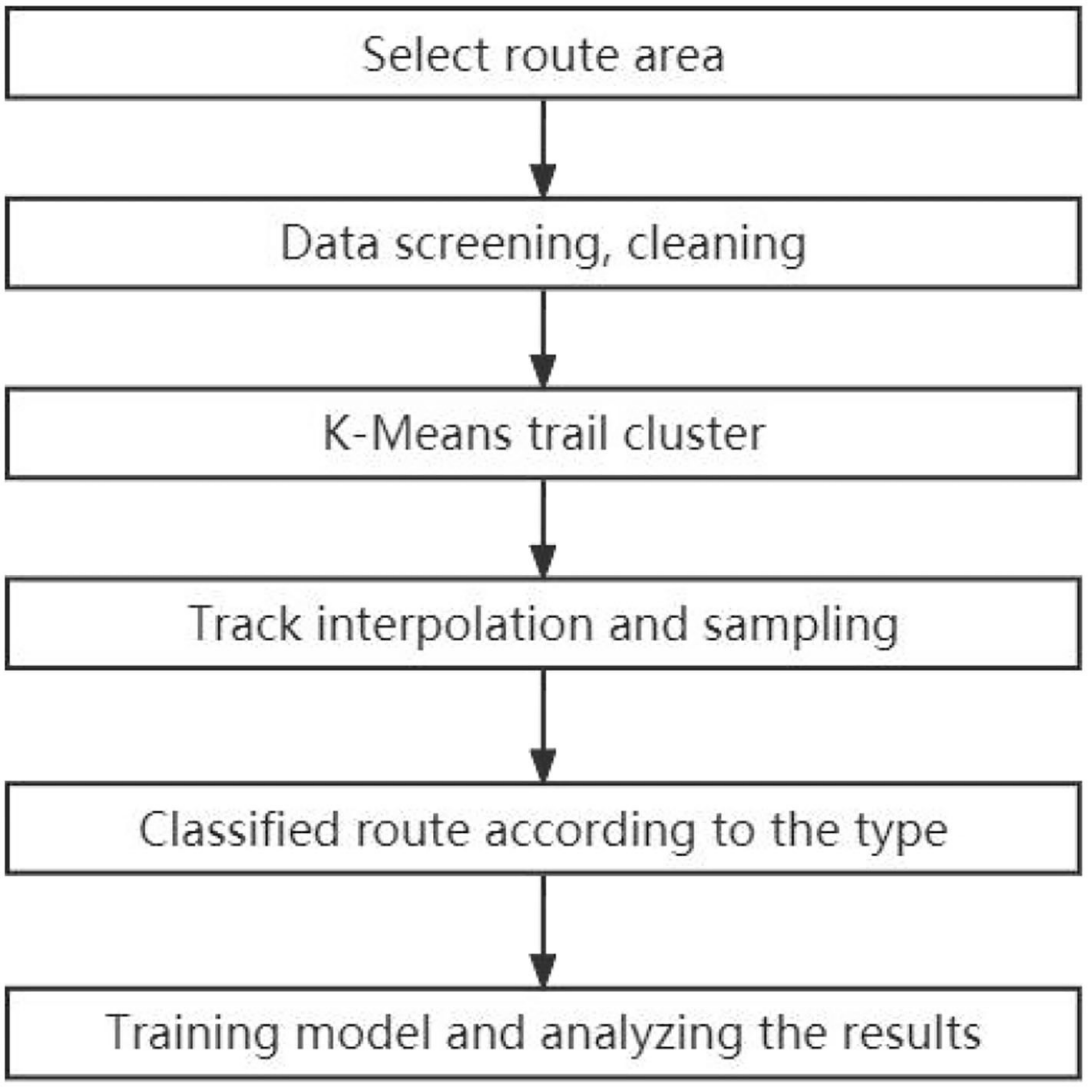
Flowchart of the route generation process.

The flowchart of the route generation process is shown in Figure 3.

## Production of datasets

### Data cleaning

**Figure 4:**
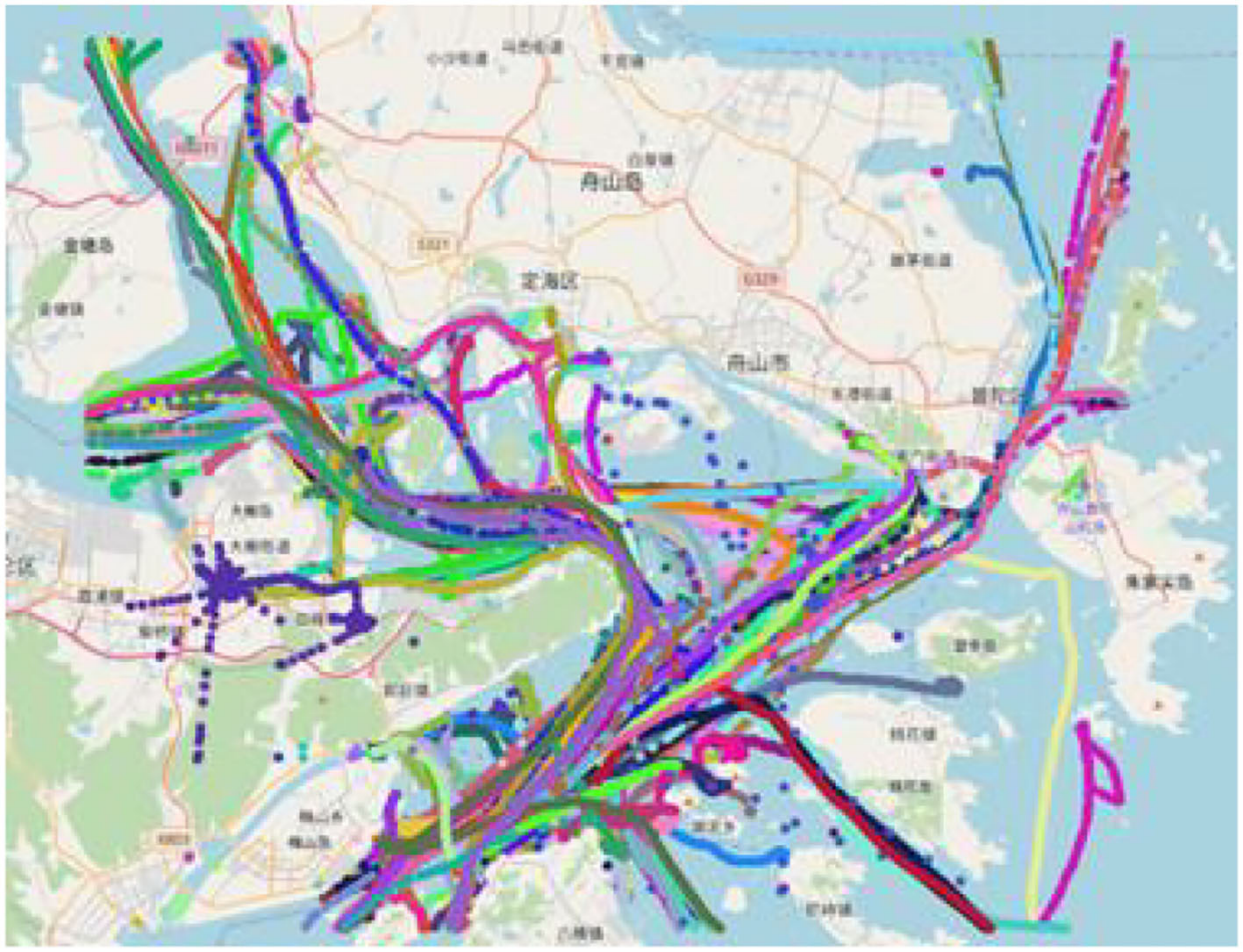
Trajectory chart of a certain day in Dinghai Sea.

The original AIS data are chaotic, with noise and a large amount of invalid data, as shown in Figure 4. Before clustering the routes, the data also need to be cleaned. The cleaning mainly includes the cleaning of abnormal data and redundant data and is listed as follows:

1. The MMSI number is not nine digits.

2. Latitude exceeds the range of 0–90°, longitude exceeds the range of 0–180°, or heading value is 511 which is a meaningless number, or the speed value is 0.

3. The state of the ship is at anchor or stationary.

4. Data move too far in a short period of time.

### K-Means algorithm for clustering

**Figure 5:**
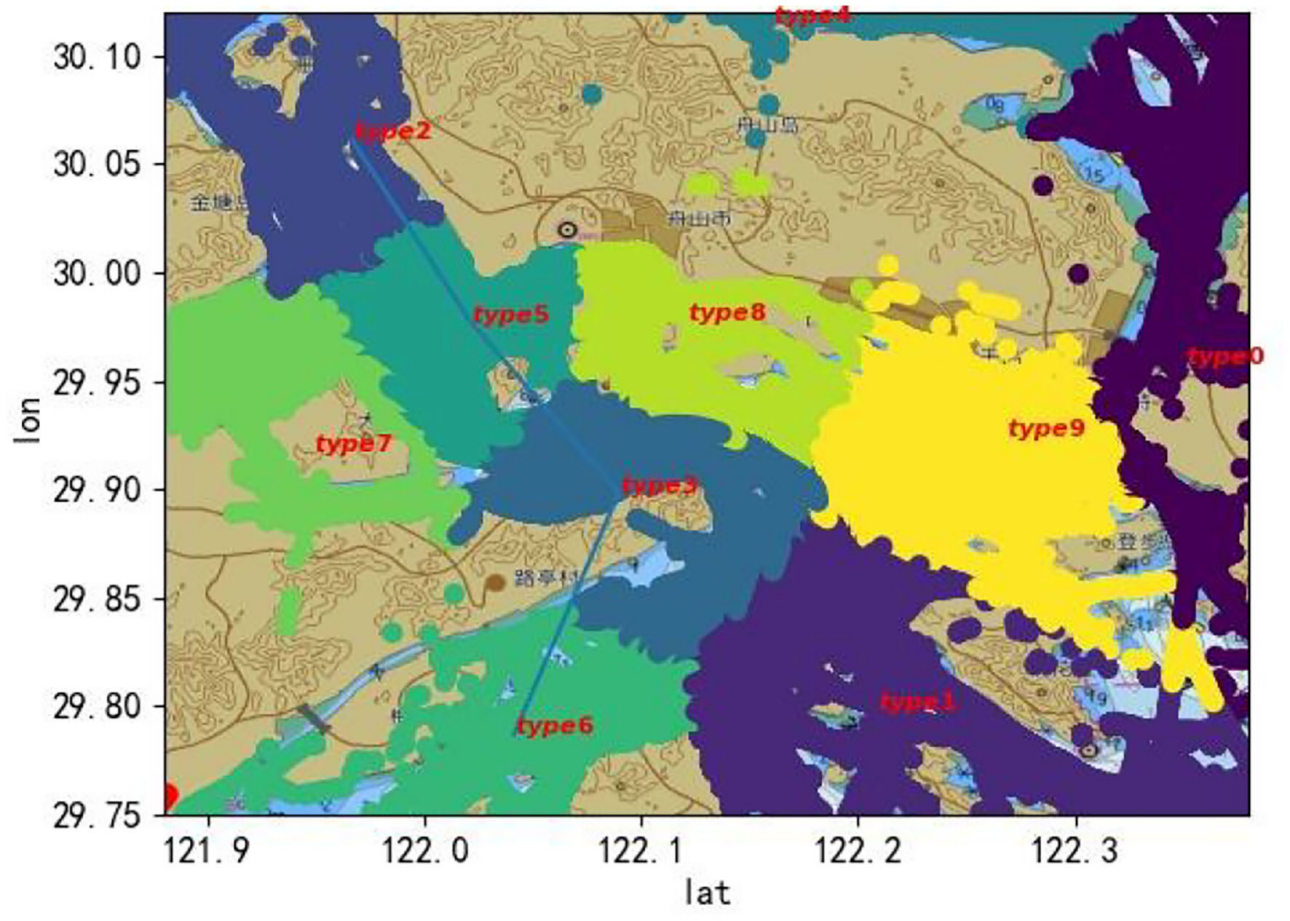
Clustering map of ship trajectories of a certain day in Dinghai Sea.

Figure 5 shows the clustering results of the ship trajectories of a certain day in Dinghai Sea. It can be seen that most of the divided clusters fall on each channel in the sea, which is due to the relatively dense trajectory points in the channel, resulting in the distance between the trajectory points in the region being close, and the center of mass also tends to fall in the channel. For clusters without fairways, the internal distances tend to be bigger and the shape of the cluster is more narrower. From Figure 5, we can see that clusters 0, 1, 4, 7, 8, and 9 are irrelevant clusters and should be removed from the route data. After the analysis of the reserved routes, it is found that the ships involved in the routes are mainly cargo ships, tankers, and container ships, which means that these three ships are the representative ships of the region, so this work will design routes for these three ship types.

### Interpolation and sparseness of trajectories

According to IMO RESOLUTION MSC.74(69) (Fujii et al., 2019), the AIS sending frequency of the route is related to the speed and heading. In addition, the LSTM requires the input data to be standard equal-length data. In the process of actual cleaning of the routes, it is found that the AIS data lengths of the routes are far different, some route data are too sparse and there are long missing values in the middle, which are not enough to extract the route features, while some route data are too dense, and the value provided by some data points is not high, which is a great waste of computing resources. To preserve the features of the route to the maximum extent, this study interpolates and samples the trajectories and compares the trajectories of different lengths. Before interpolation, it is necessary to distinguish whether the route is sparse or missing, and Table 1 lists the information on sending interval of Class A AIS equipment according to IMO RESOLUTION MSC.74 (69):

**Table 1:** Class A AIS message sending interval.

**Vessel travel status**	**Sending interval**
Ship is anchor	3 min
Ship speed 0–14 knots and constant course	12 s
Speed 0–14 knots and course change	4 s
Speed 14–23 knots and constant course	6 s
Speed 14–23 knots and course change	2 s
Speed > 23 knots and constant course	3 s
Speed > 23 knots and course change	2 s

According to Table 1, the number of missing values of routes can be calculated. If the route is sparse or the lack of data is not much, it can be interpolated. First of all, the scope of the lack of trails needs to be defined. When the absolute value of the heading difference near the missing value is within 5°, it is determined as a straight line trajectory; otherwise, it is a curve trajectory (Gao et al., 2021). After calculating the number of missing values according to Table 1, routes with straight continuous missing values between 0 and 5% of the total route length and curved continuous missing values between 0 and 2% of the route were interpolated, and trajectories exceeding these criteria were rejected (Vinisha and Sujihelen, 2022). As the linear interpolation algorithm has high operating efficiency, the route distance designed in this study is relatively short, and the routes are relatively smooth, it is suitable to use linear interpolation for implementation (Huang et al., 2011). Suppose the first two points of interpolation are (*t*_*m*_, *p*_*m*_) and (*t*_*n*_, *p*_*n*_), (*t*_*i*_, *p*_*i*_) is the data to be interpolated, where *t*_*i*_ denotes the current time stamp and *p*_*i*_ represents the ship's latitude, longitude, speed, and heading at the current time point, and then *p*_*i*_ can be expressed as


(20) $$\begin{array}{l} p_{i}\;=\;p_{n}+\frac{\left(p_{n}-p_{m}\right)}{\left(t_{n}-t_{m}\right)}\left(t_{i}-t_{m}\right) \end{array}$$

**Figure 6:**
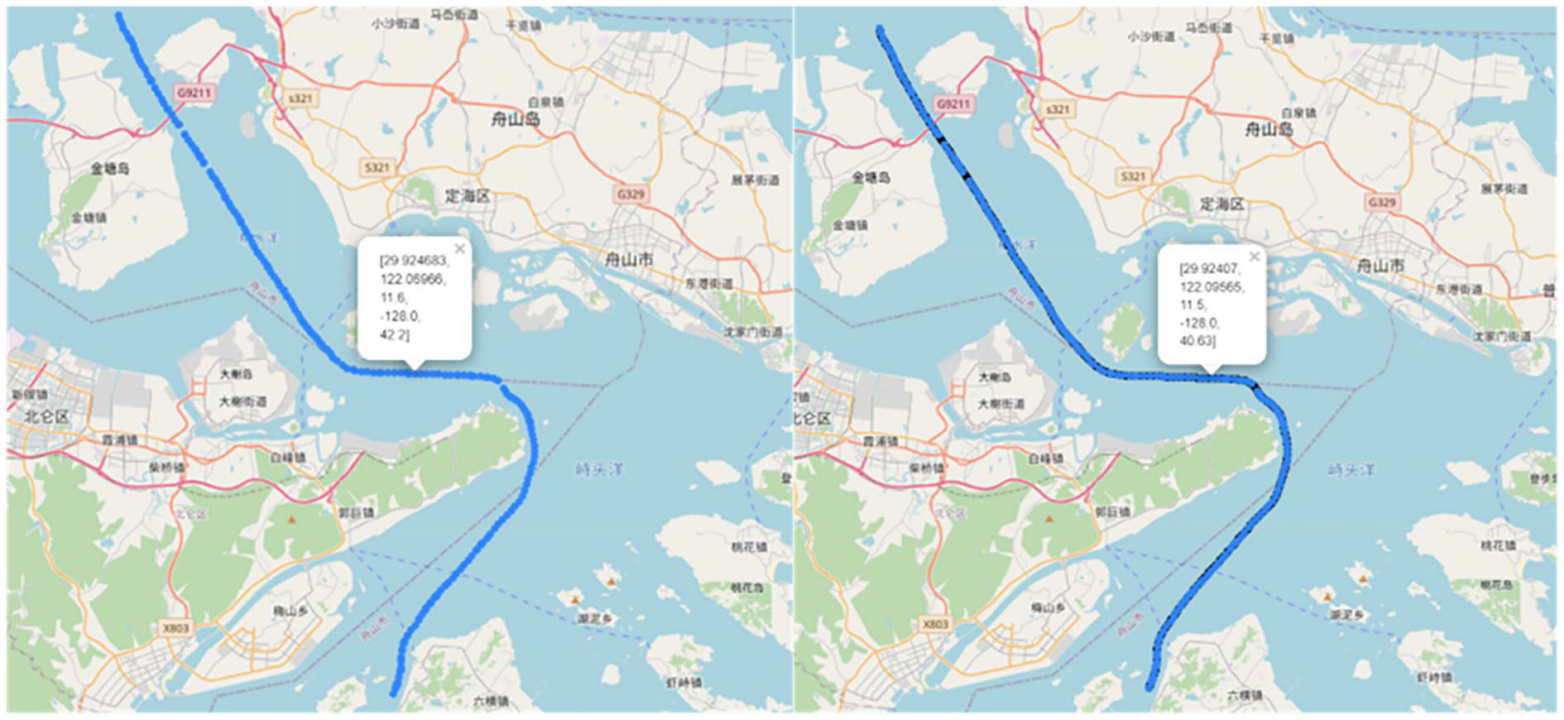
Ship trajectories before and after interpolation.

Figure 6 shows the trajectory before route interpolation on the left and the trajectory after route interpolation on the right.

Considering the characteristics of the trajectory samples are “dense trajectories of single sample and small total number of samples”. Therefore, this study uses a random mean sparse method (Huang and Zhu, 2022) to spare the high-density trajectories into multiple routes with fixed length every time, which reduce the trajectory density and increase the number of trajectory samples. The purpose of this study is to dilute the trajectory length into four lengths, 100, 200, 400, and 800, respectively. On the one hand, the purpose of this study is to reduce the error caused by interpolation, generally speaking, the longer the trajectory is, the greater the possibility of missing values, and the more interpolation is needed, so the moderate dilution of the trajectory can reduce the error. On the other hand, many trajectories cannot be used because the span of continuous missing values is too long. To increase the data sample, the continuous missing values become less after moderate dilution so as the available trajectory data are increased.

### Normalization of data

There are differences in attributes and units in the trajectory points, and when calculating the model, the data needs to be normalized to facilitate the calculation and training of the model. In this study, min-max normalization is used to process the AIS data with the following equation:


(21) $$x\;\prime \;=\;\frac{x-min(x)}{\max(x)-min(x)}$$

where x represents the data before normalization and *x*′ represents the data after normalization, followed by the normalization of the data, which is used to accelerate the convergence rate of the model weight parameters with the following equation:


(22) $$x\prime =\frac{x-x}{\sigma }$$

where


(23) $$x=\frac{1}{n}\sum_{i-1}^{n}x_{i},\sigma =\sqrt{\frac{1}{n-1}\sum_{i-1}^{n}{\left(x_{i}-x\right)}^{2}.}$$

## Route generation

### Model construction

**Figure 7:**
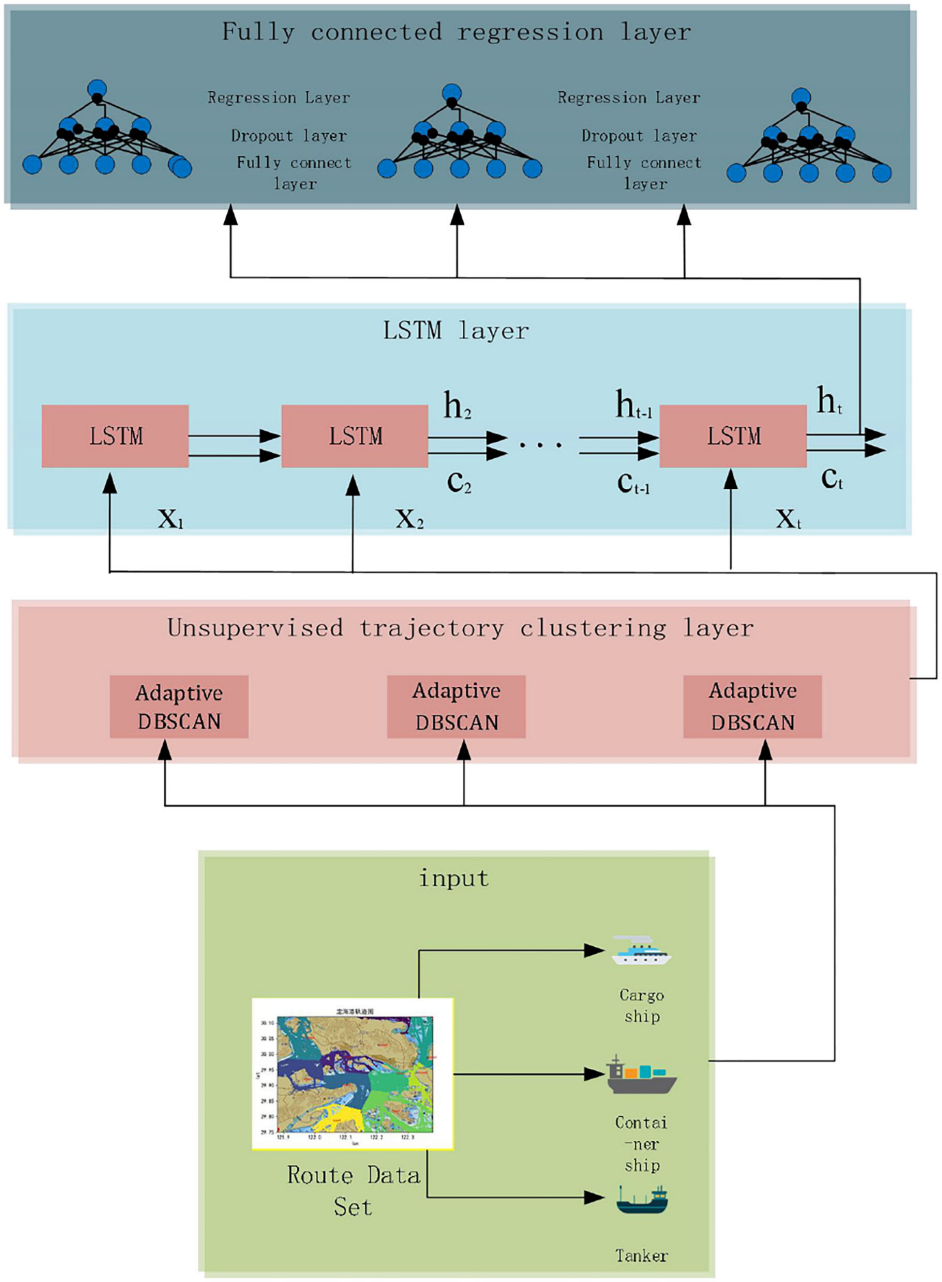
Adaptive LSTM network structure.

This experiment is mainly developed based on the python language using the Pytorch framework. The compilation software is Pycharm and Jupyter Lab, and the operating system is Ubuntu. The training process of using Pytorch to build an LSTM network mainly includes the following steps: generate sequence data; divide training set, validation set, and test set; normalize it; determine the network structure layers; and select the corresponding optimizer, activation function, and loss function, which is the main construction process of LSTM. In this study, an unsupervised trajectory clustering layer is defined on top of the LSTM model, its main role is to integrate adaptive DBSCAN into the LSTM, and the input data are automatically classified into trajectories based on the similarity of the trajectory of the ship. For the trajectory of the ship with <3,000 datasets, the sample size is too small to ensure the error of the generated route within a reasonable range. Therefore, this study sets 3,000 trajectory data samples as the threshold and only takes the route with more than 3,000 samples of each ship for training. The structure of the adaptive LSTM neural network is shown in Figure 7.

**Figure 8:**
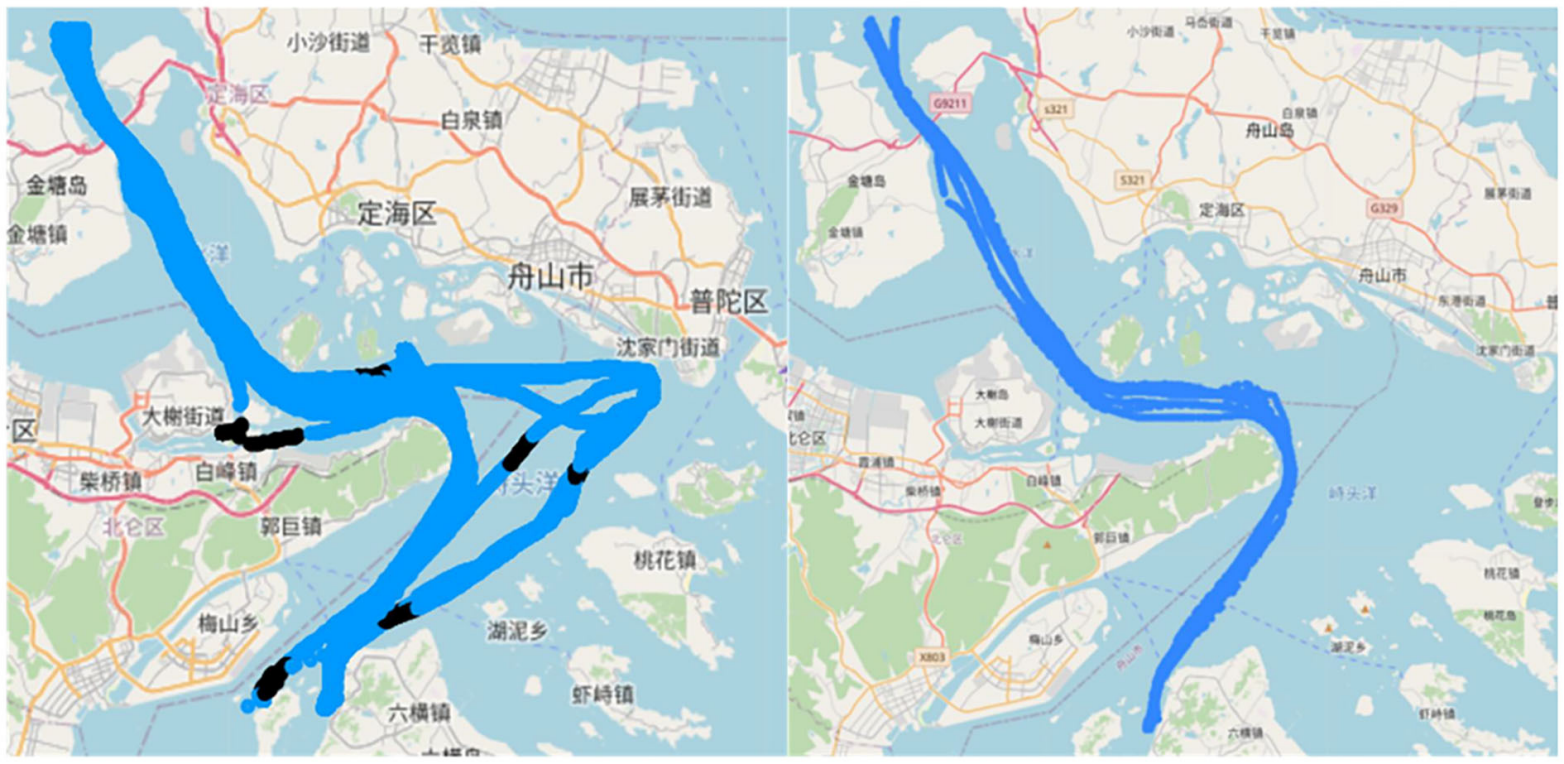
Route separation by path.

First, the input routes were classified according to ship types, and the classified data were input to the unsupervised clustering layer. Each ship's data were processed using an unsupervised clustering neuron, all the navigation trajectories in the area of that type of ship were obtained, and each path route data were separated. Figure 8 shows the route data separation process, and the routes with black dots indicate that they do not belong to the cluster.

The routes with more than 3,000 samples in the route path dataset are transferred to the LSTM layer, and each route contains four features, namely, longitude, latitude, speed (SOG), and heading (COG), which are trained separately. LSTM layer consists of four LSTM stacks, which are used to learn the route features, and the hidden state of each layer is set to 600. A fully connected layer is connected for each ship trajectory. The fully connected layer is followed by the dropout layer to prevent model overfitting. The parameter is set as 0.2, and the result is output through linear regression. In the experiment, the learning rate is 0.001, the batch size is 64, the number of iterations is 400, and Adam is used as the optimizer. Notably, 60% of the dataset is used for training, 20% for validation, and 20% for testing. The mean absolute error (MAE) and RMSE are chosen for the loss functions.

### Results and evaluation

During the experiment, it is found that the length of the trajectory also has an influence on the prediction results. In this study, it is considered that when the trajectory length is lower than 100, it is not enough to fully characterize the ship's motion information, and when the trajectory length exceeds 800, the ship's motion information will overflow.

From Table 2, it can be seen that the error of the improved LSTM is reduced compared with the original LSTM, and the error decreases by 0.01–0.03 on average when using MAE and RMSE evaluation. After comparing the prediction accuracy of different lengths of trajectories, it was found that the longer the trajectory, the larger the error, mainly due to two reasons, one is the degradation of the model performance due to the large input and output scales, and the other is that the longer the route, the more the interpolation, resulting in an increase in error. It is also found that the improved LSTM is better than the LSTM when the predicted length is 100–200, and not necessarily when the length is more than 200. Five items of the LSTM are better than the improved LSTM, indicating that the improved LSTM prediction decreases faster when the length of the trajectory grows. The error of COG and SOG is larger than that of latitude and longitude. After analysis, it is believed that the prediction range of COG and SOG is larger, resulting in a higher loss value, and the error of the model is larger because of more missing values of COG and SOG.

**Table 2:** Error comparison of different trajectory lengths and different models.

**Trajectory length**	**Feature**	**LSTM**		**Adaptive LSTM**	
		**MAE**	**RMSE**	**MAE**	**RMSE**
100	Longitude	0.013277	0.074095	**0.010324**	**0.047461**
	Latitude	0.014565	0.068520	**0.010869**	**0.045931**
	COG	0.021355	0.091246	**0.018327**	**0.073722**
	SOG	0.023546	0.085233	**0.017546**	**0.065511**
200	Longitude	0.015329	0.062920	**0.012339**	**0.059245**
	Latitude	0.012496	0.062457	**0.012319**	**0.056924**
	COG	0.024317	0.085332	**0.020315**	**0.079856**
	SOG	0.021365	0.090227	**0.021335**	**0.071183**
400	Longitude	0.017241	**0.059824**	**0.013255**	0.068548
	Latitude	0.015312	0.073946	**0.012703**	**0.067906**
	COG	0.026750	0.093240	**0.025416**	**0.089685**
	SOG	**0.021362**	0.103741	0.022693	**0.090050**
800	Longitude	**0.015766**	0.088312	0.016899	**0.088865**
	Latitude	0.019624	**0.090315**	**0.014079**	0.093658
	COG	0.025846	**0.101320**	**0.024932**	0.104418
	SOG	0.029627	0.132548	**0.024062**	**0.092673**

Bold values indicate the smaller value of the two algorithms.

**Figure 9:**
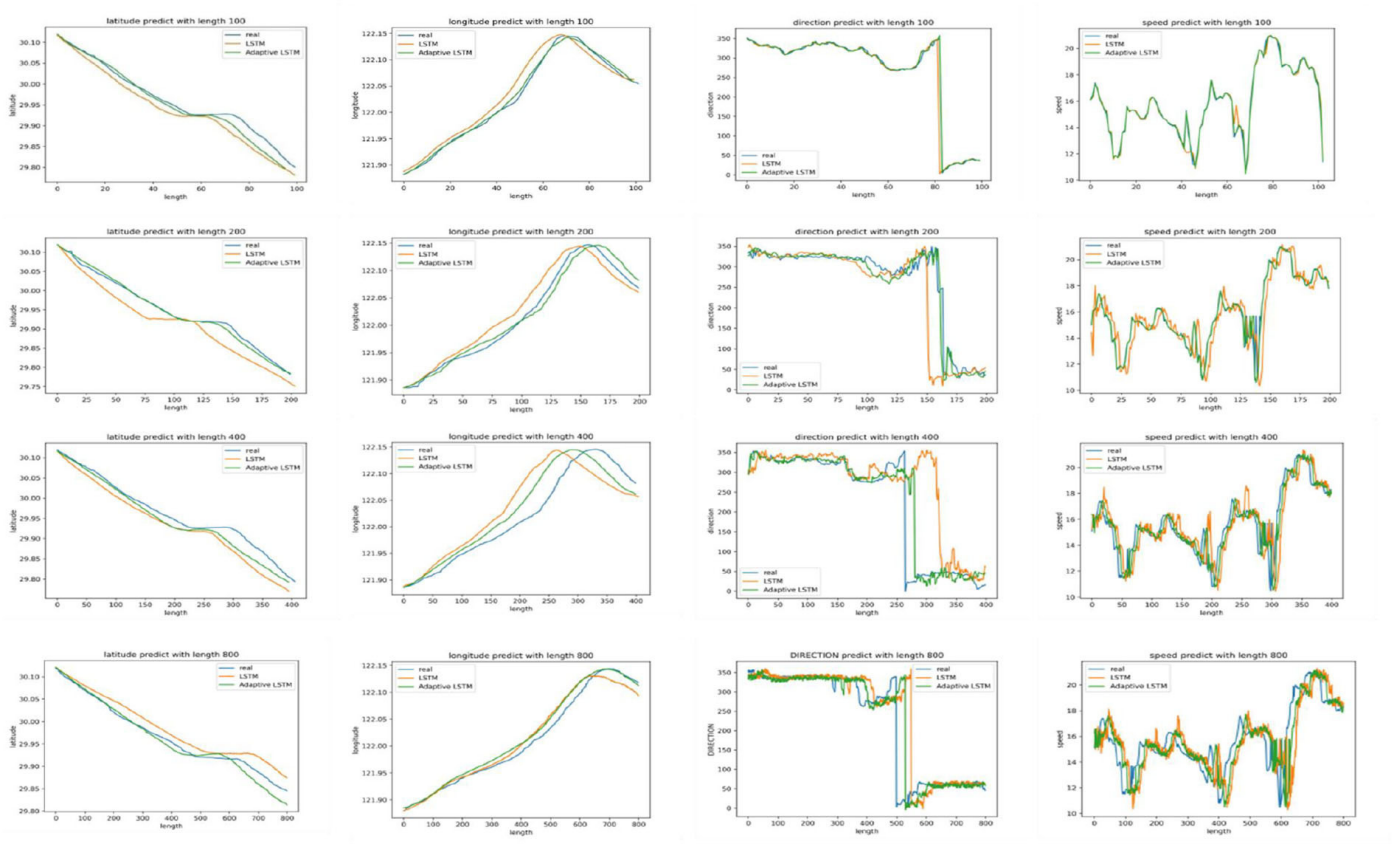
Prediction of route data with different lengths.

Figure 9 shows the prediction accuracy of the route data under different lengths. It can be seen that the improved LSTM for latitude and longitude predicts better compared to the LSTM when the trajectory length is 100 to 200, and the error of latitude and longitude is below 0.001° on average, the prediction error of COG is below 9°, and the error of SOG is around 0.1 knot on average, which are all in the acceptable range. When the length exceeds 200, the improved LSTM in this study has less improvement in prediction accuracy relative to the LSTM. The overall trend of latitude value in the route prediction data shows a continuous decline, and the value of a segment tends to be parallel, indicating that the ship is moving laterally. The overall trend of longitude value shows a rise and then a decline, indicating that the ship is turning. The COG first rises and falls in a small range and continues to rise to 0° when the ship's direction approaches 360°. In the prediction of SOG, although the prediction accuracy is high when the trajectory length is short, the rise and fall of speed are too steep, while the steepness trend of SOG decreases with the longer trajectory length.

**Figure 10:**
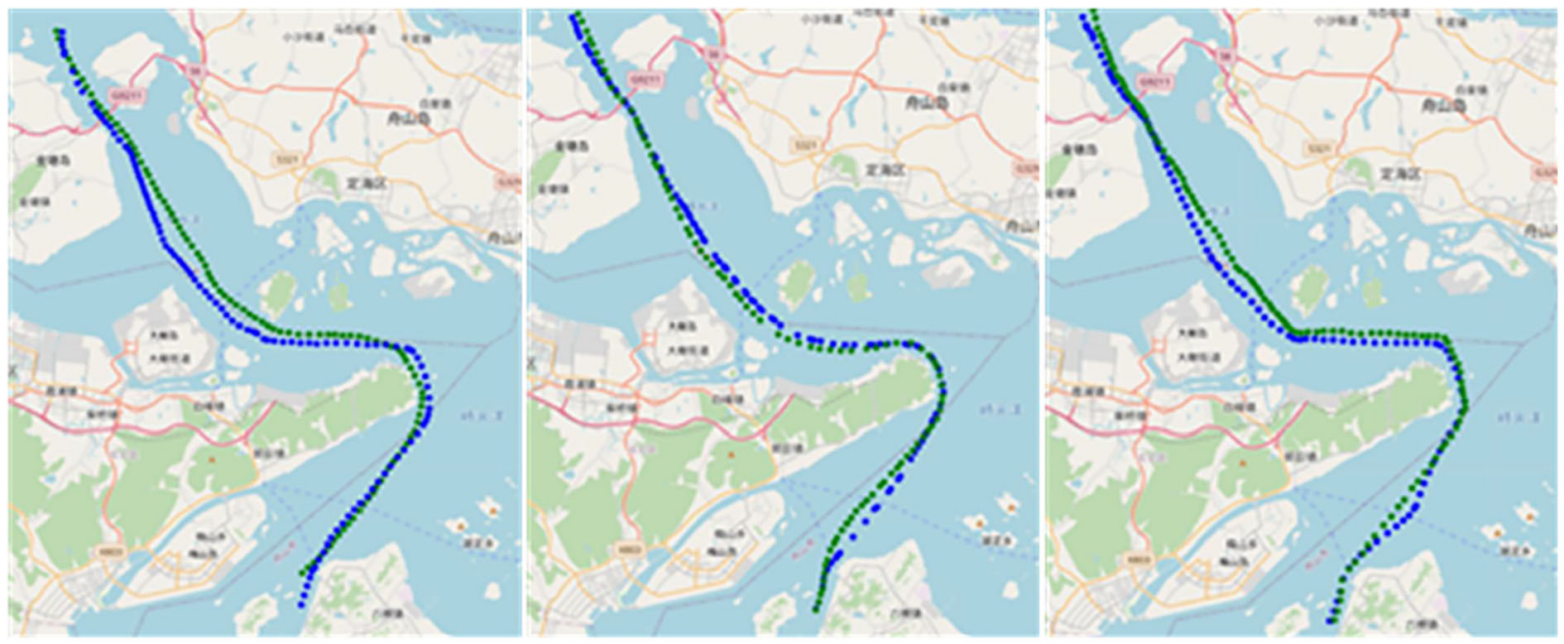
Generated routes. Blue is the actual route, green is the generated routes, and from left to right, the types are cargo ships, tankers, container ships.

Figure 10 shows the actual situation of cargo ships, tankers, and container ships on the chart when the predicted length is 100–200; it can be seen that the error with the actual route is within the acceptable range, and the predicted route is shorter and smoother in terms of trajectory length compared with the actual route, which indicates that the model has the ability to extract the route features from the dataset. Therefore, this study concludes that when the trajectory points are short, the improved LSTM generates better routes than LSTM.

## Conclusion

This study implements an autonomous route generation technique using the historical trajectory of a ship combined with an improved LSTM and discusses the comparison of the prediction error between the LSTM and the improved LSTM when the trajectory points are divided into different lengths. The results show that the prediction error of the improved LSTM is smaller than that of the LSTM when the trajectory length is smaller.

In the future, there are still many areas that need to be studied and improved. First of all, because the generation of the route relies too much on the historical trajectory of the ship, the routes with fewer ship trajectories are not considered. At the same time, due to the lack of detailed information about the ship, the factors affecting the ship's navigation are not considered enough, such as the weather and other factors are not considered. Navigational restrictions may result in routes being less practical. Second, when interpolating the missing trajectory, it does not consider whether the interpolation point will lead to the navigation restriction of the ship, which may cause the generated trajectory point to fall into the restricted area of the ship. In the following study, the abovementioned problems will be deeply studied to improve the efficiency and practicability of the autonomous route generation algorithm.

## Data availability statement

The datasets presented in this study can be found in online repositories. The names of the repository/repositories and accession number(s) can be found below: https://pan.baidu.com/s/1jQ43GF1Tqan4AAUeJ58dfQ. Further inquiries can be directed to the corresponding author/s.

## Author contributions

CC proposed the preliminary research idea of the article, provided the equipment and raw data required for the research, and revised the article after the completion of the article. CZ improved the research idea of the article and completed the experimental part of the article and the writing of the manuscript of the article. Both authors contributed to the article and approved the submitted version.

## Funding

This research was supported by the Talent Introduction Scientific Research Fund project of Zhejiang Ocean University under Grant No. 11185090821 and the Postgraduate Education Quality Series Project of Zhejiang Ocean University under Grant No. 1118106412204.

## Conflict of interest

The authors declare that the research was conducted in the absence of any commercial or financial relationships that could be construed as a potential conflict of interest.

## Publisher's note

All claims expressed in this article are solely those of the authors and do not necessarily represent those of their affiliated organizations, or those of the publisher, the editors and the reviewers. Any product that may be evaluated in this article, or claim that may be made by its manufacturer, is not guaranteed or endorsed by the publisher.
